# Effect of Sterilization on the Dimensional and Mechanical Behavior of Polylactic Acid Pieces Produced by Fused Deposition Modeling

**DOI:** 10.3390/polym15153317

**Published:** 2023-08-06

**Authors:** Israel Garnica-Bohórquez, Viviana R. Güiza-Argüello, Clara I. López-Gualdrón

**Affiliations:** 1 Industrial Design Department, Universidad Industrial de Santander, Bucaramanga 680002, Colombia; clalogu@uis.edu.co; 2 Metallurgical Engineering and Materials Science Department, Universidad Industrial de Santander, Bucaramanga 680002, Colombia; vivragui@uis.edu.co

**Keywords:** additive manufacturing, material extrusion (MEX), polylactic acid (PLA), sterilization

## Abstract

To successfully implement additive manufacturing (AM) techniques for custom medical device (MD) production with low-cost resources, it is imperative to understand the effect of common and affordable sterilization processes, such as formaldehyde or steam sterilization, on pieces manufactured by AM. In this way, the performance of low-risk MDs, such as biomodels and surgical guides, could be assessed for complying with safety, precision, and MD delivery requirements. In this context, the aim of the present work was to evaluate the effect of formaldehyde and steam sterilization on the dimensional and mechanical stability of standard polylactic acid (PLA) test pieces produced by fused deposition modeling (FDM). To achieve this, PLA samples were sterilized according to the sterilization protocol of a public hospital in the city of Bucaramanga, Colombia. Significant changes regarding mechanical and dimensional properties were found as a function of manufacturing parameters. This research attempts to contribute to the development of affordable approaches for the fabrication of functional and customized medical devices through AM technologies, an issue of particular interest for low- and middle-income countries.

## 1. Introduction

In recent decades, technological advances have led to the development of customized medical devices (MD, see Table 1), which are intended to provide functional and esthetic restoration to patients affected by trauma, injury, or pathologies in different parts of the body, including the musculoskeletal system [1]. To be used in a surgical scenario, the MD must undergo sterilization, a process that can have detrimental effects on MD integrity, and, therefore, must be carefully chosen to prevent MD failure [2]. In the 1950s, with the emergence of plastic materials for medical applications, different low-temperature sterilization techniques were developed, which promoted microbial inactivation without MD physical alterations [3]. Examples of these are (1) sterilization with ethylene oxide (C_2_H_4_O) at concentrations between 200-450 mg/L, temperatures from 37–66 °C, and cycles between 1–6 h, and (2) plasma sterilization with hydrogen peroxide (H_2_O_2_), which is carried out at temperatures of around 37–44 °C, concentrations of 6 mg/L, and 50 to 70 min cycles. More recently developed sterilization techniques include gamma [4] and ultraviolet irradiation [5]. The first one can penetrate intricate areas within the MD but can potentially induce chemical degradation of polymeric materials; on the other hand, UV irradiation is more affordable, although it only provides partial sterilization of the device [6].

From a financial viewpoint, the abovementioned sterilization techniques often require expensive specialized equipment [7]. In the context of developing countries, such as Colombia, where public hospitals most commonly have access to steam sterilization (121–132 °C) and formaldehyde sterilization (65–80 °C) systems [8], the use of high-temperature sterilization methods is essentially unavoidable. Its affordability and fast sterilization cycles make steam sterilization the preferred approach when dealing with reusable metal MDs or plastic MDs fabricated from highly resistant materials, such as polyether ether ketone (PEEK) [9] or polyetherimide (PEI) [10].

In terms of manufacturing processes, MDs are standardly produced in long series by subtractive manufacturing (SM) processes such as turning, numerical control machining (CNC), casting, or injection [11,12]. Titanium alloys (Ti_6_Al_4_V), polyethylene (PE), and polymethylmethacrylate (PMMA) have been employed as the base materials to produce sutures, fixings, bone cement, and implants through SM techniques. Nonetheless, SM technologies fail to be the most effective approach for custom MD fabrication due to geometric limitations [13], cost overruns [14], as well as material and energy waste [15]. To overcome these obstacles, additive manufacturing (AM) has emerged as a sustainable production alternative for customized MDs. Particular attention has been focused on the use of fused deposition modeling (FDM) due to its inexpensive nature, as well as the fact that it allows both the extrusion of a wide range of polymers, such as PLA [16,17], and the fabrication of highly complex structures [18]. The latter becomes key to surgical scenarios that require patient-specific devices [16,19]. In this sense, from cranioplasty implants [20] and screw guides for spine surgery [21] to chest wall reconstruction devices [22], FDM has been successfully tested in the clinical setting. Likewise, FDM-printed PLA anatomical models have been evaluated for effective preoperative planning, yielding promising results for cranial and spinal surgery [23,24].

Despite these encouraging outcomes, the implementation of AM technologies for the creation of customized MDs remains hindered by significant challenges that are primarily related to the high cost of medical-grade materials, as well as the software and hardware that support its application, which in turn limits the possibilities for technological assimilation in developing countries [25,26]. In the case of Colombia, regular medical insurance coverage (around 5000–6000 USD per patient per year [27]) would not be nearly enough to afford custom MD fabrication through 3D printing [28,29]. As a result, local orthopedic surgeons are often limited to using two-dimensional (2D) radiography as a guiding tool for surgical procedures, despite the great level of uncertainty and error it can bring [30]. In the best-case scenario, when financial resources are available, surgeons resort to foreign providers to obtain customized MDs. This solution implies high costs [31,32], long delivery times, and sometimes, poor MD fitting due to either the patient’s biological changes or partially fulfilled MD design requirements [33,34]. Therefore, in most cases, outsourcing custom MDs can become an extraordinarily inefficient and exhausting process for the medical system.

In the face of these facts, increasing attention has been drawn to the use of more affordable, FDA-approved materials for FDM processing, such as polylactic acid (PLA) [35,36] and nylon [37], which have been reported for the manufacture of MDs for orthopedic implants and scaffolds. Despite their low cost and biocompatibility, PLA and nylon-based MDs produced by FDM are still susceptible to physical and dimensional damage upon exposure to high-temperature steam sterilization [38]. Therefore, to comply with custom MD fabrication by AM standards [39,40] and fulfill MD cleaning and sterilization requirements [41,42], the dimensional stability of the polymeric piece is key to ensuring proper fitting and mechanical performance during the surgical procedure (surgical guides) or after surgery (implants). Despite the relevance and enormous potential of FDM for medical applications, especially in low- and middle-income regions, the issue of MD dimensional and mechanical stability after sterilization has been scarcely discussed. In the case of PLA, the chemical stability of the polymer can be compromised by steam autoclave sterilization [43], whereas gamma irradiation approaches can induce PLA degradation when using the recommended doses for effective sterilization (20–30 kGy) [44]. Thus, to successfully implement FDM for custom MD production with economic resources, such as PLA, it is imperative to understand the impact of common and affordable sterilization processes, such as formaldehyde or steam sterilization, on the properties of the manufactured devices [45].

In this way, the performance of MDs, including biomodels and surgical guides, could be assessed towards complying with safety, precision, and MD delivery requirements [46], as well as improving control over fabrication parameters (orientation, and infill percentage, for example [47]), which are also crucial for an accurate computer design process (CAD-CAE-CAM) [48,49]. In this context, the aim of the present work was to evaluate the effect of formaldehyde and steam sterilization on the dimensional and mechanical stability of standard PLA test pieces produced by FDM using different fabrication parameters. To achieve this, PLA samples were sterilized according to the sterilization protocol of a public hospital in the city of Bucaramanga, Colombia. This research attempts to contribute to the development of affordable approaches for the fabrication of functional and customized medical devices through FDM technologies, an issue of particular interest for low- and middle-income countries. 

## 2. Materials and Methods

**Figure 1:**
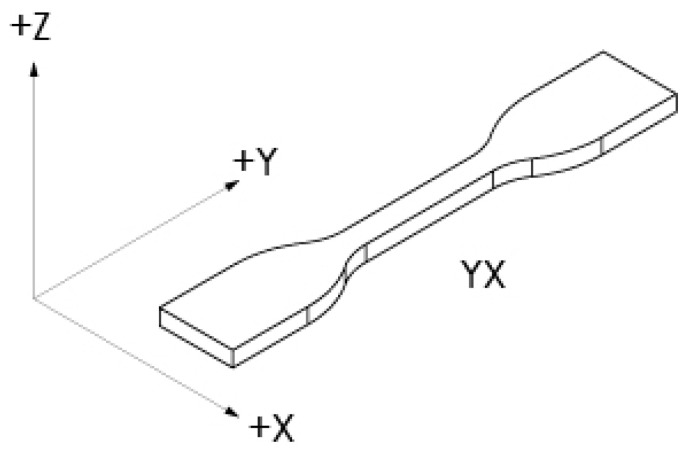
Machine coordinate system. Source: image based on ASTM [53].

Test specimens were designed using CAD software (SolidWorks Education 2020-2021, Dassault Systemes, Vélizy-Villacoublay, France) and segmented for AM by means of free software (CURA 4.6.1, Ultimaker, Utrecht, The Netherlands). A computer with Intel^®^ Core™ i5-4590 CPU @ 3.30 GHz, 16 GB of RAM, and a 64-bit Windows 8.1 operating system (Optiplex 9020, Dell, Round Rock, TX, USA) was also used. Based on the reports by Hernández et al. [50] and Afrose et al. [51], the highest performance of extruded fibers occurs in the YX direction, following the provisions of ASTM F2971-13 [52]. Thus, the machine coordinate system and sample orientation were defined as fixed variables to reduce the number of pieces to be manufactured, as illustrated in Figure 1. 

**Figure 2:**
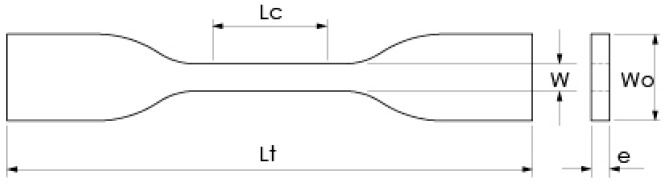
The geometry of type IV specimen. (Lc) Nominal rated length, 25 mm. (Lt) Total nominal length, 115 mm. (W) Nominal minor width, 6 mm. (Wo) Nominal width, 19 mm. (e) Nominal thickness, 4 mm. Source: based on ASTM [53].

Sample geometry for the stress test was selected based on ASTM D638-14 (type IV specimen), as shown in Figure 2 [53]. The selected type of specimen allowed for the comparison of material stiffness between sterilized samples and their corresponding control group (non-sterilized samples). PLA filament (1.75 mm diameter, esun3d, Shenzhen, China) was chosen as the printing and Supporting Material, and its properties are described in Table 2.

In terms of the 3D printing process, the following variables were defined for all samples: (1) wall thickness: 0.8 mm; (2) layer thickness in the z-axis: 0.2 mm; (3) printing speed: 40 mm/s; (4) extrusion temperature: 210 °C; and (5) printer bed temperature: 40 °C. A raft-type printing base was also included to improve piece adhesion onto the printer bed. These parameters were configured in the slicing software CURA (4.6.1, Ultimaker, Utrecht, The Netherlands) to generate the geometric code (G-Code) file for each sample group.

### 2.1. Experimental Design and Data Collection

A mixed two- and three-level 3^1^-2^1^ experimental design was used. The first factor was sterilization, with two levels: sterilized and non-sterilized. The second factor was infill percentage, with three levels: 30%, 60%, and 90% infill [56]. A total of 6 treatments were obtained to determine the possible dimensional or mechanical resistance changes for sterilized PLA pieces relative to their non-sterilized counterparts [57]. By printing 3 samples per treatment, a total of 18 specimens were manufactured without any fabrication issues, deformations, or adhesion problems between layers. [56]. An FDM open-source device (BGC Smart Tech, Voxel3d, Bogotá, Colombia) was used to build the specimens. Machine specifications were defined as follows: 300 × 300 × 300 mm for build volume; 0.4 mm for extrusion nozzle; 0.1 mm for z-axis resolution; and an aluminum build platform covered with adhesive tape (Scotch-Blue # 2090, 3M, Saint Paul, Minnesota, USA) as the build sheet. Six samples were printed for each type of filling density.

Moreover, PLA sample sterilization was performed following the sterilization protocol of a public hospital in the city of Bucaramanga, Colombia. For each type of infill, 3 specimens were subjected to sterilization, whereas the remaining 3 specimens were used as the control group (without sterilization). Sterilized samples were initially exposed to formaldehyde steam sterilization (640 autoclave, CISA, Lucca, Italy) at 16 mg/L and 65 °C through a 2 h sterilization cycle. Subsequently, samples were rested for a week at room temperature to remove any formaldehyde traces, following a second treatment via steam sterilization (Ritter M11 Autoclave, Midmark, Versailles, OH, USA) for 30 min at 132.2 °C. Sample dimensions were measured after sample fabrication as well as at the end of the sterilization process. Wo, W, and e values (Figure 2) were collected using an analog micrometer (Ubermann, Santiago, Chile), while *Lt* was measured with a digital calibrator (Ubermann, Santiago, Chile). Similarly, sample mass was measured using a digital scale (AS220.R2, Radwag, Miami, FL, USA).

Additionally, the cost and time required for PLA sample fabrication were estimated based on the building time on the FDM equipment and the amount of material that was used [58]. Although there are different cost models associated with AM [59], direct costs are generally considered derived from raw material consumption, whereas indirect costs are related to 3D printer usage (energy, maintenance, and equipment depreciation). The costs associated with human labor were omitted [60], as well as the sterilization costs, the latter because final parts are usually delivered by the manufacturer in an unsterilized condition. The data used to calculate sample manufacturing costs are summarized in Table 3.

Furthermore, sample mechanical properties were evaluated via tensile tests in a universal mechanical tester (Model Bionix, MTS, Eden Prairie, MN, USA), following ASTM D638-14 and using a constant strain rate of 3.75 mm/min [53]. Mechanical data were analyzed according to Askeland and Wright [61]. The unit strain percentage Ԑ (mm/mm) was obtained from the division between the calibrated length (Lc) and the elongation data. Also, the stress σ (N/mm^2^) was calculated from the ratio between the force and the initial cross-sectional area (W × e), as depicted in Figure 2. Based on these data, it was possible to obtain the following sample properties: Young’s modulus (E, MPa), yield stress (Sy, MPa), tensile strength (Su, MPa), and elongation percentage (elong) or elongation ductility [62].

### 2.2. Statistical Analysis

Data statistical analysis was performed employing the R Studio software (V 4.3.1, R Foundation, Vienna, Austria). Analysis was divided into two sections: The first section, using the Kruskal–Wallis test to compare non-parametric groups [63]. This method requires the verification of four assumptions: (1) independence of variables, which can be verified by Pearson’s correlation for non-parametric data; (2) dependent variables must be continuous; (3) a normal data distribution is not required; (4) variance between groups must be homogeneous, a condition that is verified through Bartlett’s test or Levene’s test. The Kruskal–Wallis test was followed by Dunn’s test to identify statistical differences between treatments. A portion of the R code that was used is shown below in Listing 1 (describing comments in italic).

The second section used hierarchical clustering analysis and principal component analysis (PCA) to obtain similarities among normalized data to identify factors that summarize most of the variation [64]. A portion of the R code that was used is shown below in listing 2 (describing comments in italic). Find the complete code for Listing 1 and 2 in the dataset [65].

**Listing 1.** Nonparametric comparison code.*#to import data* [66].
install.packages(“readxl”)
library(readxl) 
Z <- read_excel(“dir/data.xlsx”,col_names = TRUE)
*#to name factors; sterile, infill, Treatment.*

Z$sterile <- as.factor(Z$sterile)
Z$sterile =factor(Z$sterile,labels= c(“control”,”sterile”)) 
*#assumption 1. Correlation among variables* [67].
> corz <- cor(Z, y=NULL, method = “pearson”)
round(cor2z,2)
*#assumption 2. Variables must be continuous.*
*#assumption 3. Non-parametric data. To group treatments.*

Group1 <- subset(Z,Tr==“Control-Infill-30%”)
…
Group9 <- subset(Z,Tr==“Sterile-infill-90%”)
*#variable Lt Behavior in group 1.*
qqnorm(Group1$Lt)
qqline(Group1$Lt)
*#assumption 4. Homogeneous variance among groups* [68]. 
install.packages(“car”)
library(car)
*#Variance among treatments related to Lt variable* [69].
leveneTest(Z$Lt ~ Z$Tr, Z = Z)
install.packages(“FSA”)
library(FSA)
*#Kruskal-Wallis’ test and Dunn’s Test for Lt Vector variable respect to treatments column Tr.*

Lt <- c(Z$Lt)
kruskal.test(Lt,Z$Tr)
dunnTest(Lt,Z$Tr,method=“bonferroni”)

**Listing 2.** Multivariable analysis code.*#to make a data copy.*
Z <- datapla2
*#to disable factors columns (sterile, infill, Treatment) into the dataset.*
datapla2$Tr <- NULL
*#to normalize data. Data range between 0 and 1 for dimensionless comparison* [70]. 
set.seed(250) #to make the results reproducible
data.norm <- rnorm(nrow(datapla2)) *# to shuffle rows using normal distribution.*
datapla2 <- datapla2[order(data.norm),] *#data reorganization by the vector.*
normalize <- function(x){
+ return((x-min(x))/(max(x)-min(x)))} *# to define function.*
Data.N<-as.data.frame(lapply(datapla2[,c(1,2,3,4,5,6,7,8,9,10,11,12)], normalize)) *#to apply the normalize function in data.*
#libraries [71,72,73,74]
library(factoextra)
library(cluster)
library(ggplot2)
library (stats)
*#clustering data with hierarchical method* [64,75,76]
*#to define linkage methods m*
 m <- c(“average”, “single”, “complete”, “ward”)
 names(m) <- c(“average”, “single”, “complete”, “ward”)
 #function to compute agglomerative coefficient
 ac <- function(x) {agnes(data.N, method = x)$ac}
 sapply(m, ac) *# calculate agglomerative coefficient near to 1.*
*#to calculate number of clusters k vs gap statistic, iterations B ≥ 500.*
gap_stat <- clusGap(data.N, FUN = hcut, nstart = 25, K.max = 10, B = 500)
fviz_gap_stat(gap_stat) *#results depend on the biggest jump in within-cluster distance after uniformity.*
*#distance matrix calculation.*
res.dist = dist(x = data.N, method = “euclidean”)
*#hierarchical method.*
res.hc <- hclust(d = res.dist, method = “ward.D”)
*# Cluster dendrogram.*
fviz_dend(x = res.hc, cex = 0.7, lwd = 0.7)
*# Principal component analysis PCA plot.*
fviz_cluster(object = list(data=data.N, cluster=cutree(res.hc, k=5)))
*# to determine cluster by sample.*
g <-cutree(res.hc, k=5)
table(g)
g_pla <- cbind(data.N[,-1],g)
print(g_pla)

## 3. Results

### 3.1. Data Collection

**Figure 3:**
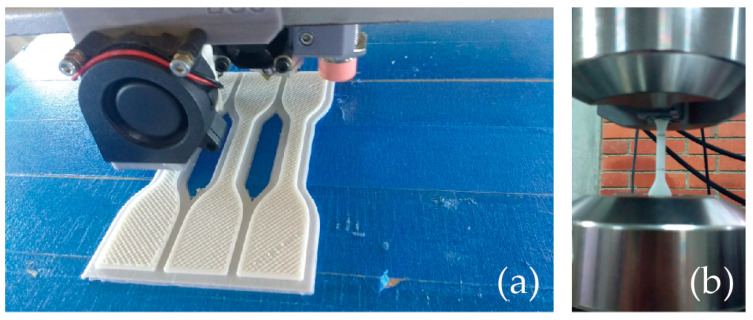
PLA samples: (**a**) building procedure, (**b**) tensile test setup.

PLA samples were 3D printed by nesting groups of three specimens at the same time, centered at the building origin (see Figure 3), using an average printing time of 1 h ± 10 min per specimen. Table 4 shows the collected sample data in detail. All samples were subjected to tensile testing. The experimental setup for the tensile tests can be found in Figure 3.

**Figure 4:**
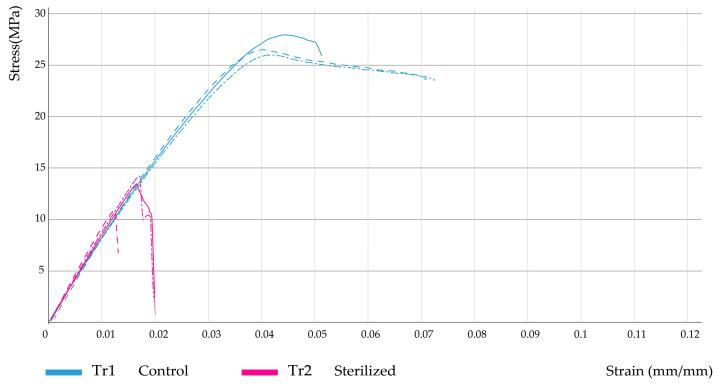
Stress–strain curves for 30% infill samples.

**Figure 5:**
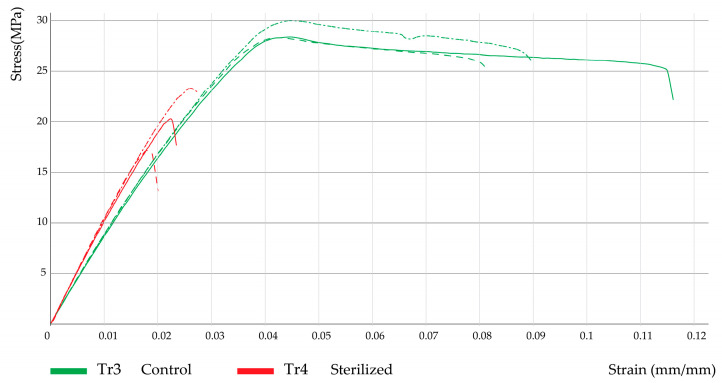
Stress–strain curves for 60% infill samples.

**Figure 6:**
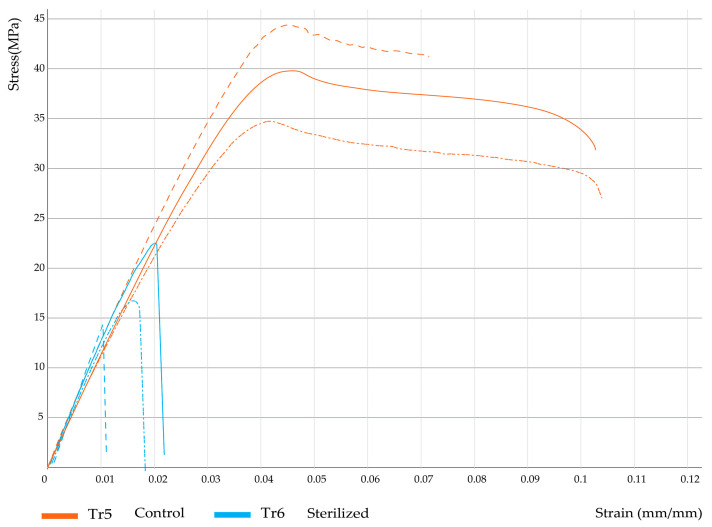
Stress–strain curves for 90% infill samples.

**Figure 7:**
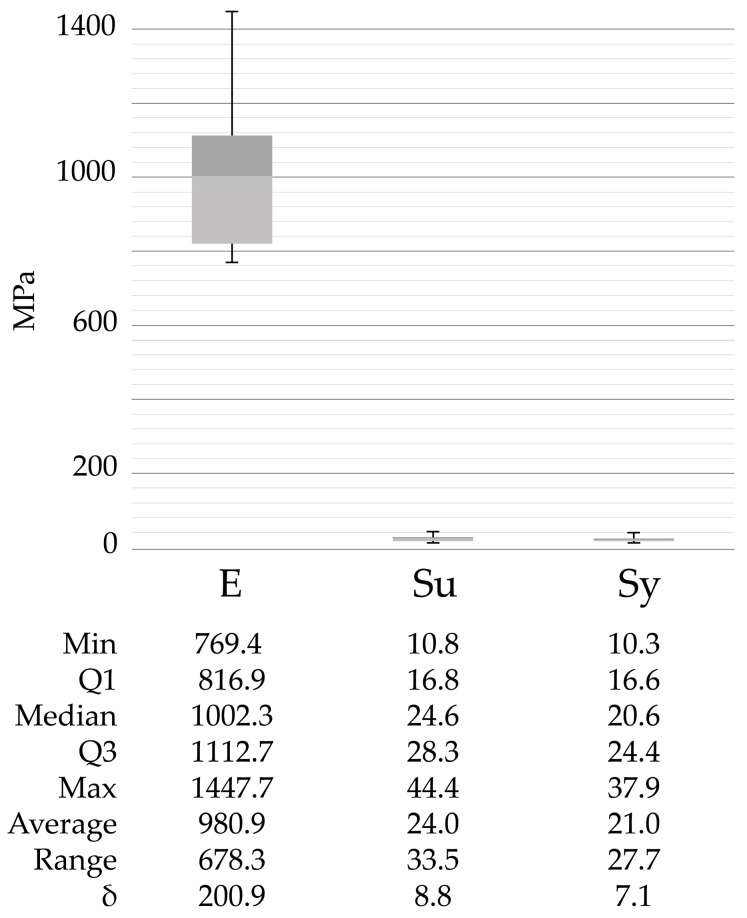
Data distribution for all sample groups that were evaluated. Min (Minimum), Q1 (1st Quartile), Q3 (3rd Quartile), Max (Maximum), δ (Standard deviation), E (Young’s modulus), Sy (Yield Strength), Su (Ultimate strength).

Furthermore, Figure 4, Figure 5 and Figure 6 show the corresponding stress/strain curves for each of the filling density groups (30%, 60%, and 90% infill). For all groups, a notable decrease in mechanical resistance was observed in the sterilized samples relative to their non-sterilized counterparts. Moreover, a comparison of the control groups for the 30% and 60% infill samples suggests similar yield strength values, although the toughness appears to be greater for the 60% samples, as evidenced by the enhanced plastic zone. Moreover, Figure 7 presents the data distribution for all tested samples, which indicated significant changes and asymmetric distributions for Young’s Moduli data (980.9 ± 200.9 MPa), although strength values appeared much less variable across groups: Sy (21.0 ± 7.1 MPa) and Su (24.0 ± 8.8 MPa).

### 3.2. Nonparametric comparison

Table 5 shows the results from the Kruskal–Wallis test, which allowed us to confirm statistical differences among samples with two levels of significance: *p*-value < 0.05 (highlighted in yellow) and *p*-value < 0.01 (highlighted in green). Statistical differences were found for all variables except for W. 

Following the Kruskal–Wallis test, Dunn’s test was applied to determine exactly which groups were statistically different. First, the dimensional variables Lt, Wo, and e were evaluated. Table 6 shows the results from pairwise comparisons between each independent group. It is important to remember here that groups TR1 (30% infill), TR3 (60% infill), and TR5 (90% infill) correspond to the non-sterilized PLA samples (controls), while treatments TR2, TR4, and TR6 represent their corresponding sterilized PLA counterparts. Comparison 1 corresponds to treatments TR1 and TR2, described in Figure 4; comparison 10 corresponds to treatments TR3 and TR4, as seen in Figure 5, and comparison 15 corresponds to treatments TR5 and TR6, as seen in Figure 6.

The results in Table 6 show that for comparison 1 (groups TR1 and TR2), no significant statistical differences were found, but for comparison 10 (groups TR3 and TR4) and comparison 15 (groups TR5 and TR6) differences regarding sample length and thickness were significant, which indicated that the sterilization process had an important effect on the dimensional stability of PLA samples manufactured at 60% and 90% filling density, respectively. In addition to this, when evaluating the effect of filling density on the dimensional behavior of the PLA pieces, significant differences were found in terms of thickness for the 30% infill samples (sterilized and non-sterilized) relative to the 90% infill samples (TR5). On the other hand, when comparing all sterilized groups (TR2, TR4, TR6), no significant changes were identified in terms of dimensional variables. 

Furthermore, Table 7 presents the results from Dunn’s test for the mechanical properties of the different PLA samples under study. 

As suggested by the stress-strain curves previously shown, several significant differences were found between groups: comparison 1 (groups TR1 and TR2, 30% infill) indicated significant differences in terms of ultimate strength (Su); for comparison 10 (groups TR3 and TR4, 60% infill) significant changes were identified for Young’s modulus (E) and % elongation (elong); also, for comparison 15 (groups TR5 and TR6, 90% infill) significant differences in terms of yield strength (Sy), ultimate strength (Su), and percentage elongation (elong) were found. Moreover, comparison across filling densities (30%, 60%, and 90% infill) indicated significant changes in Young’s modulus between the 30% and the 90% infill groups, regardless of the presence of the sterilization procedure, since for the sterilized groups (TR2 and TR6) a *p*-value of 0.04 was found, whereas for the non-sterilized groups (TR1 and TR5) a *p*-value of 0.05 was obtained. Cumulatively, the results from Table 6 and Table 7 indicated that, for a specific filling density, the sterilization process had a significant effect on the dimensional and mechanical behavior of the manufactured PLA pieces. 

### 3.3. Multivariable Analysis

Data in Table 3 were normalized to facilitate comparison among dimensionless variables. Because some scales and values are larger than others, normalization reduces this dominance. Thus, it was possible to reduce the influence of outliers in the data by modifying the data scale and preserving normal distribution. To observe patterns of similarities across the data, clustering analysis allows data reduction and outlier identification. The most common methods are k-mean, k-medoids, and hierarchical methods [64]. The last one was selected because it does not depend on the number of clusters beforehand; it is most reliable for the identification of outliers and preserves distance information between small data. A hierarchical cluster first calculates the distance between observations. Then it fuses the most similar data into a cluster and assigns a group until it finishes comparisons. Different methods could be selected for determining data closeness. To calculate dissimilarity, the agnes() function in R returns the agglomerative coefficient from 0 to 1, meaning a robust clustering if this coefficient is near 1. Table 8 shows these results.

The selected agglomerative coefficient indicated the ward’s linkage method selection. This method minimizes variance, starting on individual data points until merging with a cluster, then successively shaping a hierarchy of clusters. To choose the number of clusters (k), another metric named gap statistic was used. It determines from 0 to 1 the optimal k comparing the total inter-clustering variation (sum of squared distances) of a given clustering solution with a non-apparent cluster structure (random) in the dataset [75]. 

**Figure 8:**
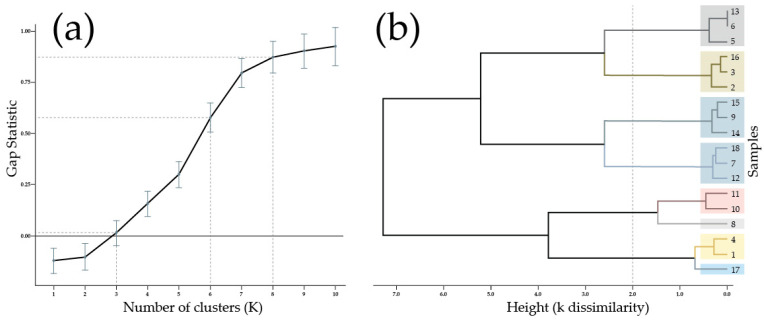
Hierarchical clustering: (**a**) gap statistic between 0 to 1 for k clusters up to 10. (**b**) dendrogram for k = 8 clusters and samples’ number corresponding with Table 3. Dash line indicated the cluster analysis for determine a suitable k value.

The optimal gap statistic calculated with clusGap() and fviz_gap_stat() functions in R from the normalized dataset was k = 8, being a near-to-zero value of k = 3, as can be seen in Figure 8a. To make a dendrogram, it was necessary to calculate a square matrix from data dissimilarity by Euclidian distance [76]. With the previous information, it was possible to run hierarchical clustering for k = 8 with the hclust() function in R, as shown in Figure 8b. It could be observed that clustering highlighted samples 8 and 17 as outliers. However, depending on how the height is selected, different clusters could be obtained with acceptable dissimilarity. For example, if the chosen height is 2, the k clusters are 6, with a gap statistic above 0.5 that could fit. This example was indicated by dash line in Figure 8a,b.

**Figure 9:**
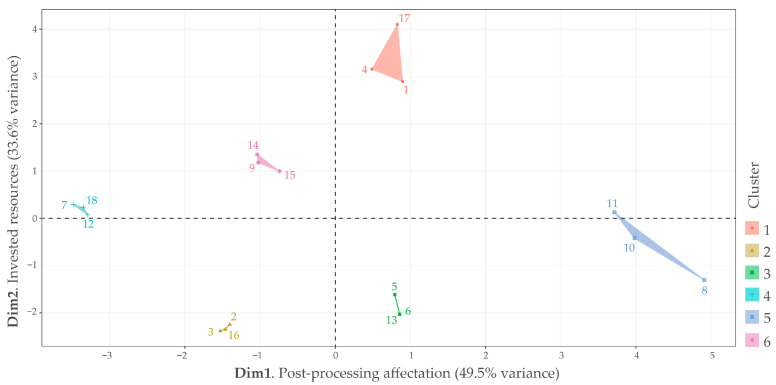
Principal components reduction (PCA) from hierarchical clustering k = 6 clusters. Colors 1 to 6 correspond to each cluster. Euclidian distance. Linkage method Ward.D. Numerical data from 1 to 18 represent each sample.

Additionally, it was observed that hierarchical clustering did not group data in consistency with treatments as initially anticipated. However, some exceptions could be seen in Figure 8b, such as the clusters that represent treatment 4, consisting of samples 10 and 11, and treatment 1, comprising samples 2 and 3. Furthermore, hierarchical clustering also revealed a hidden factor among the nine variables that could explain the behavior of the 18 samples. By PCA, the nine variables were reduced to two dimensions that accumulated 83.1% of data variance (Figure 9). Nevertheless, data among clusters were scattered with no apparent pattern, whereas PCA requires subjective analysis to identify common aspects that could fit dimension explanation. 

Dimension 1 explained 49.5% of the data variance. In the negative axis of dimension 1, 33% of samples were controlled (6 samples), and the material was 22% for 90% infill (four samples) with only 11% for 30% infill; in contrast, the positive axis of Dimension 1 had 33% of sterilized samples (six samples), and the material was 22% for 30% infill (four samples) with only 11% for 90% infill. Samples for 60% infill were equally distributed among Dimension 1 for 16% in each interval (three samples for both positive and negative axis). Because the negative axis in Dimension 1 seems stronger than the positive axis in terms of mechanical properties, according to the results from the previous subsection, that is why Dimension 1 could be named “post processing affectation”. 

Dimension 2 explained 33.6% of the data variance. In the negative axis of Dimension 2, 22% of samples were controlled and 22% sterilized (four samples each one), and the material was 22% for 30% infill, 11% for 60% infill, and 11% for 90% infill (four, two, and two samples, respectively); in contrast, in the positive axis of Dimension 2, 27% of samples were controlled and 27% sterilized (five samples each one), and the material was 11% for 30% infill, 22% for 60% infill, and 22% for 90% infill (two, four, and four samples, respectively). The similarity in the sterilization factor indicated that this factor did not seem to influence this dimension. Instead, infill percentage could be a better explanation because in the positive axis in Dimension 2, it concentrates the samples with the most mass as well as manufacturing time. That is why Dimension 2 could be named “invested resources”. 

The multivariable data analysis indicates that it could be possible to explain data in terms of affectation for sterilization related to processing resources. For example, samples 7, 8, 9 (non-sterilized with 60% infill) and 10, 11, 12 (sterilized with 60% infill) are spread across Dimension 1 but near to Dimension 2’s origin, which could explicate their preference as affordable and practical manufacturing parameters.

## 4. Discussion and Conclusions

In the context of the Sustainable Development Goals, global surgery constitutes a key element in achieving global health and social equality. In developing countries, access to adequate surgical care is predominantly hindered by financial constraints [77]. According to the Lancet Commissions, the unmet surgical needs for low- and middle-income countries worldwide were estimated at about 143 million annual procedures in 2015 [78]. To further complicate this issue, the incidence of conditions such as traumatic spinal injury or spine degenerative disease appears to be significantly higher in low- and middle-income countries relative to high-income countries [79,80]. If performed timely, these injury/disease-related surgical treatments could prevent death or disability. However, the lack of essential surgical supplies frequently becomes one of the most delaying factors. 

Additive manufacturing approaches have emerged as promising tools to address the abovementioned shortcomings. Among these technologies, fused deposition modeling has drawn special attention due to its inexpensive nature and the fact that it allows both the extrusion of a wide range of polymers, such as PLA [16,17], as well as the fabrication of highly complex structures [18]. The latter becomes key to surgical scenarios that require patient-specific devices. In this sense, from cranioplasty implants [20] and screw guides for spine surgery [21] to chest wall reconstruction devices [22], FDM has been successfully tested in the clinical setting. Likewise, FDM-printed PLA anatomical models have been evaluated for effective preoperative planning, yielding promising results for cranial and spinal surgery [23,24].

The primary goal of the present studies was to evaluate the effect of formaldehyde and steam sterilization on the dimensional and mechanical stability of standard PLA test pieces produced by FDM toward contributing to the development of low-cost approaches for the fabrication of functional and customized medical devices. Combining affordable manufacturing processes with engineering materials, such as PLA, is of particular interest to the medical community, especially in low- and middle-income contexts, since, for example, the production of PLA-based 3D printed cranial models could cost anywhere between 5–150 USD [81,82]. Specifically for Colombia, utilizing PLA offers multiple advantages, as it has been demonstrated that local PLA production from natural resources is economically feasible due to Colombia’s unique biodiversity as well as its large stock of agro-industrial waste [83,84,85]. In this sense, fused deposition modeling stands out as a cost-effective AM technology that would enable the use of such locally produced PLA for in-house medical device fabrication. 

Nonetheless, several challenges must be overcome before FDM can be successfully implemented as a reliable and economical alternative for MD production in contexts where financial resources are particularly limited. For instance, MD dimensional and mechanical stability after sterilization is one of the key requirements that remain to be consistently fulfilled. Preservation of geometric and structural properties after sterilization is crucial to ensuring both the accuracy of the biological fitting as well as the adequate mechanical performance of a PLA-based medical device. The early work of Neches et al. [86] attempted to circumvent this issue by studying whether the temperature and pressure used for extrusion in FDM were enough to guarantee PLA sterilization. The authors manufactured 52 specimens inside a laminar cabinet with and without UV light. Their results from in vitro testing indicated a 10% risk of sample contamination, a number that would be unacceptable in any clinical setting, especially if the fabricated MD is to be used inside the human body. 

To gain insight into the set of processing parameters that predominantly affect MD dimensional and mechanical integrity, we set out to evaluate the effect of formaldehyde and steam sterilization on standard PLA pieces produced by FDM according to the sterilization protocol of a public hospital in the city of Bucaramanga, Colombia. Relative to methods such as ethylene oxide sterilization or plasma sterilization with hydrogen peroxide, formaldehyde, and steam sterilization are more readily available in public hospitals due to their economic nature. In 2012, Perez et al. [87] reported their findings from a study aimed at evaluating the effect of different sterilization techniques (autoclave, ethylene oxide, hydrogen peroxide, and gamma radiation) on the sterility of polymeric parts produced by FDM. Acrylonitrile butadiene styrene (ABS), a polymer of comparable commercial cost as PLA, was one of the studied materials. Although their experiments were not focused on the analysis of dimensional behavior, they reported that physical damage was observed for the ABS samples after autoclave and flash autoclave treatment. 

Our results evidenced that sterilization of 3D-printed standard PLA pieces significantly affected several dimensional and mechanical parameters, such as sample length, thickness, Young’s modulus, yield strength, ultimate strength, and elongation percentage. Moreover, when analyzing the impact of filling density, it was found that the dimensional and mechanical behavior of the 30% infill group was significantly different from that of the 90% infill group. Although our experiments were designed using the minimum number of specimens that allowed statistical power [61], it was possible to draw relevant conclusions that will guide future studies, which will include greater sample size and, thus, higher statistical power. 

The thermoplastic nature of low-cost polymers such as PLA or ABS poses one of the major challenges to the achievement of MDs that can withstand high-temperature sterilization conditions. Nonetheless, the results from recent studies on PLA have shown promise in alternative approaches to overcome this issue. For instance, the experiments conducted by Shaik et al. [88] attempted to elucidate the effect of pressure and temperature on the mechanical performance and consolidation of layers of 3D-printed PLA pieces. By looking at different post-production treatments (pressure + temperature, only pressure, and only temperature) in which the temperature was kept at the glass transition (Tg) value of PLA or below, the authors were able to attain significant increases in Young’s moduli of up to 40–50%. Because at Tg the polymer chains increase their mobility, this probably helped relieved structural tension and allowed for structure reorganization that resulted in enhanced mechanical resistance. In addition, the application of high pressure aided in removing voids and maintaining isotropic properties in the PLA sample. 

Moreover, the recent work by Chen et al. [89] provided additional insight as they evaluated seven different types of commercial PLA brands, which were employed for the FDM printing of PLA samples with two geometries: cubic and standard army-navy retractor. These PLA samples were subjected to heat treatments in hot water (water bath annealing) and in a regular autoclave. Upon analysis of their results, the authors concluded that the “Essentium PLA” brand, in conjunction with a “grid” infill geometry, demonstrated a promising combination of processing parameters for the FDM fabrication of anatomical models since it provided the most dimensionally stable PLA samples.

Increasing the degree of crystallinity through annealing has been proven to endow PLA-printed pieces with high-temperature resistance since crystalline structures exhibit enhanced structural stability above the glass transition temperature. For instance, Romanov and coworkers [90] reported the development of high-pressure and heat-resistant PLA-printed microfluidic devices, which were annealed on a hot plate at temperatures between 100–140 °C after FDM printing. The authors showed that the annealed systems could withstand pressures of up to 3 MPa, as well as effectively work at 95 °C without significant deformation of the channel’s cross-section. More recently, Frizziero et al. [91] manufactured cutting guides for bone correction surgery using standard PLA, heat-treatable PLA (HTPLA), and nylon. The parts were exposed to a 50 min steam sterilization process that cycled between 70 and 134.9 °C, after which an assessment of sample dimensional stability was performed. Interestingly, deformation levels for PLA and HTPLA samples were below 1.82%, whereas nylon guides exhibited deformation levels above 2.5% [90,91].

Cumulatively, analysis of our results in the context of current literature seems to indicate that the next avenue of research to pursue could be exploring different PLA annealing conditions [89] as pre-treatment methods to avoid the detrimental effects of steam sterilization since annealing can help relieve internal tensions, as well as enhance interlayer adhesion and thermal stability of FMD-printed PLA parts. Sourcing locally synthesized PLA for such studies would also allow the simultaneous evaluation of the potential of recycled or biobased PLA as an engineering material for the fabrication of 3D-printed surgical devices, which would imply an additional opportunity for economic growth. Moreover, for example, the accurate manufacture of low-cost anatomical models would significantly help surgeons understand complex anatomical conditions prior to surgery, facilitating the provision of high-quality health care in contexts where financial resources are significantly limited.

## Figures and Tables

**Table 1:** List of Abbreviations.

Abbreviation	Description	Abbreviation	Description
δ	Standard deviation.	MPa	Mega Pascals
Ɵ	Diameter.	p	Density
Ԑ	unit strain percentage	PCA	Principal Component Analysis
σ	stress	P-A	Adjusted *p*-value
AM	Additive Manufacturing	P-Ua	Unadjusted *p*-value
ABS	Acrylonitrile butadiene styrene	PLA	Polylactic acid
ASTM	American Society of Testing and Materials.	PE	Polyethylene
Avg	Average	PEI	Polyetherimide
CAD	Computer-aided Design	PMMA	Polymethylmethacrylate
CAE	Computer-aided Engineering	PEEK	Polyether-ether-ketone
CAM	Computer-aided Manufacturing	Q1	1st quartile
CNC	Computerized Numerical Control	Q3	3rd quartile
COP	Colombian pesos	SM	Subtractive Manufacturing
E	Young’s modulus	STL	Stereolithography
e	Nominal thickness	Sy	Yield Strength
elong	Elongation at Break	Su	Ultimate strength
FDM	Fused deposition modeling	Tg	Glass transition
G-Code	Geometric Code	TR	Treatment
Lc	Nominal rated length	USD	American dollars
Lt	Total nominal length	UV-light	Ultraviolet light
Max	Maximum	W	Nominal minor width
Min	Minimum	Wo	Nominal width
MD	Medical Device	Z	Statistical value
MEX	Material extrusion	Z-axis	Perpendicular axe to the printing bed

**Table 2:** Commercial PLA filament properties. Source: based on esun3d [54,55].

Property	Value
Density (p)	1.01 g/cm^3^
Melting point	220–260 °C
Yield Strength (Sy)	62.63 MPa
Elongation at Break (elong)	4.43%
Ultimate Strength (Su)	65.02 MPa
Flexural Modulus	2504.4 MPa

**Table 3:** Estimated sample manufacturing costs. Reference prices in Colombian pesos (COP) and US dollars (USD).

Item	Unit	COP	USD
PLA filament material	g	80	0.0264
3D printer depreciation	min	9.13	0.0030
Energy consumption	kJ	0.012	3.33 × 10^−6^
Maintenance	min	2.28	0.0008

**Table 4:** PLA specimen properties with and without sterilization. From left to right: (Lt) total length, (Wo) width, (W) minimum width, (e) thickness, (E) Young’s modulus, (Sy) yield stress, (Su) ultimate strength, (elong)% elongation percentage, and cost.

Sample Identification				Dimensional Properties				Mechanical Properties				Cost USD
Sample	Sterilized	Infill %	Treatment	Lt mm	Wo mm	W mm	e mm	E MPa	Sy MPa	Su MPa	Elong %	
1	No	30	TR-1	115.2	19.1	6.3	4.0	769.4	21.5	26.1	7.4	0.39
2				115.2	19.1	6.3	4.1	794.1	20.6	27.4	5.1	0.39
3				115.4	19.1	6.3	4.1	792.8	20.2	26.0	7.2	0.39
4	Yes		TR-2	113.5	18.8	6.3	4.1	850.4	12.9	13.3	2.0	0.39
5				113.9	18.8	6.2	4.1	850.4	13.3	13.9	2.1	0.39
6				113.6	18.9	6.2	4.1	915.2	10.3	10.9	1.9	0.39
7	No	60	TR-3	115.3	19.2	6.3	4.1	792.1	24.4	28.4	11.5	0.43
8				115.3	19.2	6.3	4.1	792.1	24.4	28.4	11.5	0.43
9				115.2	19.1	6.3	4.1	820.0	25.8	30.1	9.0	0.43
10	Yes		TR-4	113.5	18.8	6.2	4.2	1028.5	19.8	20.2	2.3	0.43
11				113.5	18.8	6.2	4.1	1036.6	20.8	23.3	2.8	0.43
12				113.4	18.9	6.3	4.2	1044.0	17.1	17.2	2.1	0.43
13	No	90	TR-5	115.3	19.2	6.4	4.2	1015.7	30.5	34.8	10.4	0.49
14				115.4	19.2	6.4	4.3	989.8	29.2	33.6	6.0	0.49
15				115.5	19.3	6.1	4.4	1181.5	38.0	44.4	7.2	0.49
16	Yes		TR-6	113.4	18.9	6.4	4.1	1447.7	11.7	14.3	1.1	0.49
17				113.9	18.9	6.4	4.0	1295.9	20.6	22.4	2.1	0.48
18				113.8	18.8	6.4	4.1	1241.1	16.4	16.7	1.7	0.48

**Table 5:** Kruskal–Wallis’ test results. *p*-values are highlighted in yellow (*p* < 0.05) and green (*p* < 0.01).

Kruskal-Wallis’ Test	Lt	Wo	W	e	E	Sy	Su	elong
Chi-Squared	14.09	15.18	9.28	13.96	15.72	14.77	16.27	15.43
Degree of freedom	5	5	5	5	5	5	5	5
*p*-value	0.015	0.0096	0.098	0.016	0.0076	0.011	0.0061	0.0087

**Table 6:** Dunn’s test for PLA sample dimensional variables. Z, statistical value. P-Ua, unadjusted *p* value. P-A, adjusted *p* value. *p*-values < 0.05 (yellow), and *p*-values < 0.01 (green).

Variable	Dull Test	Treatment Comparison														
		1	2	3	4	5	6	7	8	9	10	11	12	13	14	15
		TR1			TR2			TR3			TR4			TR5		
		TR2	TR3	TR4	TR5	TR6	TR3	TR4	TR5	TR6	TR4	TR5	TR6	TR5	TR6	TR6
*Lt*	Z	1.8	0.2	2.3	−0.7	1.7	−1.5	0.5	−2.4	0.0	2.0	−0.9	1.5	−2.9	−0.5	2.4
	P-Ua	0.1	0.8	0.02	0.5	0.1	0.1	0.6	0.01	1.0	0.04	0.4	0.1	0.003	0.6	0.02
	P-A	1.0	1.0	0.4	1.0	1.0	1.0	1.0	0.2	1.0	0.6	1.0	1.0	0.05	1.0	0.2
*Wo*	Z	1.9	−0.5	1.6	−1.3	1.0	−2.3	−0.3	−3.1	−0.8	2.0	−0.8	1.5	−2.8	−0.5	2.3
	P-Ua	0.1	0.6	0.1	0.2	0.3	0.02	0.8	0.002	0.4	0.04	0.4	0.1	0.005	0.6	0.02
	P-A	0.9	1.0	1.0	1.0	1.0	0.3	1.0	0.03	1.0	0.6	1.0	1.0	0.1	1.0	0.3
*e*	Z	−0.8	−1.6	−2.3	−3.1	−0.4	−0.8	−1.5	−2.3	0.4	−0.7	−1.5	1.2	−0.8	1.9	2.7
	P-Ua	0.4	0.1	0.02	0.002	0.7	0.4	0.1	0.02	0.7	0.5	0.1	0.2	0.4	0.1	0.007
	P-A	1.0	1.0	0.3	0.03	1.0	1.0	1.0	0.3	1.0	1.0	1.0	1.0	1.0	0.9	0.1

**Table 7:** Dunn’s test for PLA sample mechanical variables. Z, statistical value. P-Ua, unadjusted *p*-value. P-A, adjusted *p*-value. *p*-values < 0.05 (yellow), and *p*-values < 0.01 (green).

Variable	Dull Test	Treatment Comparison														
		1	2	3	4	5	6	7	8	9	10	11	12	13	14	15
		TR1			TR2			TR3			TR4			TR5		
		TR2	TR3	TR4	TR5	TR6	TR3	TR4	TR5	TR6	TR4	TR5	TR6	TR5	TR6	TR6
*E*	Z	−1.1	−0.1	−2.2	−2.0	−3.1	1.0	−1.1	−0.9	−2.1	−2.1	−1.9	−3.1	0.2	−0.9	−1.1
	P-Ua	0.3	0.9	0.03	0.05	0.002	0.3	0.3	0.4	0.04	0.03	0.1	0.002	0.8	0.4	0.3
	P-A	1.0	1.0	0.4	0.7	0.03	1.0	1.0	1.0	0.6	0.5	0.8	0.03	1.0	1.0	1.0
*Sy*	Z	1.6	−1.0	0.4	−1.7	0.9	−2.6	−1.2	−3.3	−0.7	1.4	−0.7	1.9	−2.1	0.5	2.6
	P-Ua	0.1	0.3	0.7	0.1	0.4	0.01	0.2	0.001	0.5	0.2	0.5	0.1	0.04	0.6	0.009
	P-A	1.0	1.0	1.0	1.0	1.0	0.1	1.0	0.02	1.0	1.0	1.0	0.8	0.6	1.0	0.1
*Su*	Z	2.1	−0.7	0.8	−1.4	1.2	−2.8	−1.2	−3.4	−0.8	1.5	−0.7	1.9	−2.2	0.4	2.6
	P-Ua	0.04	0.5	0.4	0.2	0.2	0.006	0.2	0.001	0.4	0.1	0.5	0.1	0.03	0.7	0.009
	P-A	0.6	1.0	1.0	1.0	1.0	0.1	1.0	0.009	1.0	1.0	1.0	0.8	0.4	1.0	0.1
*elong*	Z	1.9	−1.0	1.0	−0.2	2.1	−2.9	−0.9	−2.1	0.2	2.0	0.8	3.1	−1.1	1.1	2.3
	P-Ua	0.1	0.3	0.3	0.9	0.03	0.004	0.4	0.04	0.8	0.05	0.4	0.002	0.3	0.3	0.02
	P-A	0.8	1.0	1.0	1.0	0.5	0.1	1.0	0.6	1.0	0.7	1.0	0.03	1.0	1.0	0.3

**Table 8:** Agglomerative coefficient.

Average	Single	Complete	Ward
0.843705	0.761088	0.891512	0.927438

## Data Availability

Tensile test results (Excel file) and code in the RStudio extension are available online as a dataset [65]. Supporting information can be downloaded at: https://data.mendeley.com/datasets/kf5p3yf76k/2 (V2 published on 1 August 2023).

## Supplementary Materials

The following supporting information can be downloaded at: https://data.mendeley.com/datasets/kf5p3yf76k/2 [65] (accessed on 1 August 2023).
